# Knowledge, Attitude, and Perceived Barriers of Newly Graduated Registered Nurses Undergoing Standardized Training in Intensive Care Unit Toward Early Mobilization of Mechanically Ventilated Patients: A Qualitative Study in Shanghai

**DOI:** 10.3389/fpubh.2021.802524

**Published:** 2022-01-11

**Authors:** Jinxia Jiang, Sijia Zhao, Peng Han, Qian Wu, Yan Shi, Xia Duan, Songjuan Yan

**Affiliations:** ^1^Emergency Department, Shanghai Tenth People's Hospital, School of Medicine, Tongji University, Shanghai, China; ^2^Nursing Department, Shanghai Tenth People's Hospital, School of Medicine, Tongji University, Shanghai, China; ^3^Nursing Department, Shanghai First Maternity and Infant Hospital, School of Medicine, Tongji University, Shanghai, China; ^4^Intensive Care Unit (ICU), Shanghai Tenth People's Hospital, School of Medicine, Tongji University, Shanghai, China

**Keywords:** newly graduated registered nurses, standardized training, early mobilization, mechanical ventilation, qualitative research

## Abstract

**Aim:** To explore the knowledge and attitudes of newly graduated registered nurses, who have undergone standardized training in the intensive care unit, about the early mobilization of mechanically ventilated patients and identify perceived barriers to the application of early mobilization.

**Background:** Early mobilization of mechanically ventilated patients has been gradually gaining attention, and its safety and effectiveness have also been verified. Nurses in intensive care units are the implementers of early mobilization, and the quality of their care is closely related to patient prognosis. However, the knowledge and attitude of newly graduated registered nurses undergoing standardized training, in intensive care units, on the early mobilization of mechanically ventilated patients and the obstacles they face in clinical implementation are still unclear.

**Methods:** This qualitative study utilized the phenomenological method to explore the experiences of 15 newly graduated registered nurses undergoing standardized training in intensive care units in a 3rd hospital in Shanghai, China. Semi-structured face-to-face interviews were conducted in June 2020. The Colaizzi seven-step framework was used for data analysis.

**Findings:** A total of 15 new nurses comprised the final sample after data saturation. Three main themes emerged from the analysis and seven subthemes: perceived importance, low implementation rate, and perceived barriers.

**Conclusions:** Newly graduated registered nurses undergoing standardized training in intensive care units have a high level of awareness of the importance of early mobilization of mechanically ventilated patients and are willing to implement it. However, there is a lack of relevant knowledge and other obstacles that restrict clinical implementation. Early mobilization should be included in the standardized training of new nurses in intensive care units.

## Background

Owing to the use of mechanical ventilation (MV) and monitoring equipment, as well as the insertion of various catheters, critically ill patients have to stay in bed for a long time in the intensive care unit (ICU). This makes patients prone to various physical and psychological complications, such as ICU-acquired weaknesses (ICU-AWs), deep vein thrombosis, pulmonary infection, delirium, and cognitive dysfunction (1). These complications seriously affect the short- and long-term prognoses of patients (2). Meanwhile, no specific treatments or drugs for ICU-AWs have been found so far (3). However, several guidelines state that early mobilization (EM) can improve patient outcomes (4, 5). The term EM refers to physical activity of sufficient intensity to produce physiological effects such as enhancement of blood circulation, central and peripheral vascular perfusion, ventilation, muscle metabolism, and alertness (6). Several meta-analyses have shown that EM is effective in preventing ICU-AWs, shortening the time invested in mechanical ventilation practices and stay in the ICU, and improving the body's active functions (7, 8).

EM is considered a positive intervention for ICU patients (9), but the current implementation rate of EM in ICUs worldwide is not ideal. A survey (10) in ICUs of 103 hospitals above the 2nd level in Sichuan Province, China showed that the rate of EM was 67.96%, which was slightly higher than Bakhru's research (11) in the United States. However, 58.57% of the 70 ICUs that conducted EM did not refer to or develop a standardized early mobilization program, and only 38.57% of ICUs had a dedicated team in Xie's survey (10). Tian's study (12) showed that passive and active activities in bed were frequently performed up to 50%. Bedside sitting and bedside chairs were performed more than 50% of the time. Bedside sitting was more frequently performed by 19% than bedside chair. Occasional implementation of bedside standing was about 5%. However, the frequency of bedside walking was zero. The current implementation rate of EM is still suboptimal. The clinical implementation of early activity faces difficulties such as the lack of health care professionals with relevant knowledge (13).

EM is an intervention that requires multidisciplinary cooperation (14). To ensure patient safety, the physician makes an assessment and judgment, and the rehabilitator makes an individualized activity plan based on the patient's actual condition, which the nurse assists or guides the patient to complete (15). Nurses play an important role in the implementation of EM (16). As direct participants, the quality of care provided by ICU nurses has a direct impact on patient prognosis. Meanwhile, ICU nurses' attitudes and beliefs about EM will directly influence the implementation process and effectiveness (17). Currently, ICU nurses' implementation behaviors in EM are not ideal (18), especially for newly graduated registered nurses (NGRNs). The term NGRNs refer to nurses who perform clinical nursing work within 2 years of graduation. Although NGRNs have undergone systematic learning in schools, they lack clinical experience (19). Meanwhile, NGRNs in the ICU face the working environment with critical patients, heavy resuscitation tasks, and complicated instruments and equipment. To help NGRNs adapt to the clinical environment and improve their professional capabilities quickly, the National Health and Family Planning Commission of China issued the “Training Program for New Nurses (Trial)” in February 2016 (20).

The program pointed out that the training content mainly includes basic training (basic theoretical knowledge and common clinical nursing operation-based technical training) and professional training (professional theory and practical ability training). Basic training lasts from 2 weeks to 1 month. Professional training is conducted in four different specialized departments, including internal medicine, surgery, emergency and ICU, and gynecology. Each specialty requires 6 months of training (20). Standardized training helps NGRNs improve clinical nursing knowledge and skills to a certain extent and enhances job competencies (21). However, due to the late launch of NGRNs' standardized training in China, the training system is not yet complete. A recent study (22) has shown that there is a gap between the current standardized training of Chinese clinical NGRNs and policy, such as the lack of content on completeness of training, resulting in a lack of clinical competence and critical thinking skills of NGRNs. Most of the critical care specialist's training involves the acquisition of basic theoretical knowledge and the continuous repetition and reinforcement of various nursing operations. This model cannot effectively cultivate the critical care abilities of NGRNs.

Previous studies have focused on the experiences of ICU-assigned medical staff in carrying out EM (17, 23). However, it is not clear how inexperienced NGRNs carry out EM in ICUs that require higher nursing abilities. Hence, this study aimed to explore the knowledge, attitudes, and perceived barriers of NGRNs undergoing standardized training in the ICU for EM of MV patients in Shanghai, China.

## Methods

### Study Designs and Setting

A descriptive qualitative design was used to describe the knowledge, attitudes, and perceived barriers of NGRNs undergoing standardized training in the ICU of a third level class-A hospital in Shanghai for EM of MV patients (24). A qualitative descriptive design allows exploring real-life perspectives resulting from direct experience and enables the interpretation of data for practical use (25).

### Participants

The participants were selected through purposive sampling through email and WeChat. Purposeful sampling is commonly used in qualitative research to identify and select information-rich cases related to the phenomenon of interest (26). The eligibility criteria were as follows: (1) newly graduated registered nurses in a third level hospital in Shanghai; (2) undergoing standardized training in the ICU for at least 1 month, (3) willing to participate in this study; and (4) qualified in Chinese verbal skills and able to present a reliable description of the study phenomenon.

### Data Collection

Semi-structured face-to-face interviews were conducted in July 2021. The interviews consisted of nine open-ended questions, focusing on attitudes and experiences regarding the EM of MV patients. The interview questions in Table 1, based on previous research (27), were used to explore the perception of NGRNs on the EM of MV patients in the ICU. Each interview was conducted according to the willingness and convenience of the nurses in a private and quiet meeting room. All researchers have past experiences of qualitative research. No relationship established prior to study commencement and the participants did not know the researchers personally. Before data collection, a pilot interview with two NGRNs was conducted to ensure clarity and identification of any potential problems. The pre-interviews were considered as a test only and not included in analysis. The interview started with an open-ended question which was followed by more specific questions. Each NGRN had an approximate duration of 58 min (range: 41–70 min), in which two researches participated, one of them by conducting the face-to-face interviews and the other by recording the participants' responses, including the non-verbal cues and body language during each interview. Sampling was terminated at the point of data saturation when no new information emerged (24) from the last three participants. A total of 15 new nurses comprised the final sample after data saturation. No nurses dropped out during the interviews. At the end of the interviews, each nurse was given a bottle of shampoo as a mark of appreciation.

**Table 1:** Questions for interviewing nurses about perception on the early mobilization of MV patients in the ICU.

**No**.	**Question**
1	How do you think about the early mobilization for mechanically ventilated patients?
2	What kind of early mobilization does your department carry out for patients with MV? Who will implement it?
3	What are the benefits of early mobilization for patients?
4	How to carry out risk assessment for patients before early mobilization?
5	What is the early mobilization plan of MV patients in your department?
6	What kind of training have you received on early mobilization of MV patients?
7	What do you think are the current barriers to the early mobilization in MV patients?
8	What are your suggestions on the early mobilization of MV patients?

### Ethical Consideration

Ethical approval was obtained from the Institutional Review Committee of Shanghai Hospital. Each participant signed a form with written informed consent before the interviews were conducted. Participants were informed that they were assigned codes instead of their real names featuring in the study. Participants were informed that the interviews would be audio-recorded with their permission, and their information was kept confidential and anonymous. Participation in this study was voluntary, and participants were free to withdraw at any time.

### Data Analysis

The audio recordings were transcribed verbatim and checked for accuracy by repetitive listening within 24 h of the interviews. Two experienced investigators independently analyzed the data. Data analysis was conducted beginning immediately after the first interview. The Colaizzi seven-step process for the phenomenological approach (24) was applied for data analysis. The Colaizzi seven-step includes: (1) read each statement carefully; (2) re-read, highlight and extract significant statements; (3) formulate meanings from all significant statements; (4) identify and organize the formulated meanings into theme clusters; (5) Describe the investigated phenomenon exhaustively; (6) formulate the exhaustive description to identify the fundamental structure; (7) return to the participants to confirm the findings. The authors discussed their disagreements until a consensus was reached. The transcribed data and extracted themes and subthemes were sent back to the participants and to ensure that the findings reflected the participants' actual perspectives rather than the investigators' understanding of the phenomenon, a follow-up conversation was conducted. All participants agreed to be contacted again. The interpreted themes were intended to reflect their perceptions accurately.

### Trustworthiness

Four criteria were used to assess trustworthiness: credibility, dependability, confirmability, and transferability (28). Credibility in this study was ensured through triangulation of informant participants with various working and training experiences. Observational notes and memos were written both during and immediately after the interviews, including the investigator's thoughts and feelings, impressions, as well as the interpretation of the participants' non-verbal cues and body language (29). For dependability, the investigators provided data to an experienced independent investigator to perform an independent examination of the data for confirmation. To enhance confirmability, two experienced reviewers reviewed the decision trail as well as the findings and conclusions of the study. Transferability was ensured by presenting a clear description of the study context, sampling, and the process for data collection and analysis. This research was guided by the Comprehensive Standard for Reporting Qualitative Research (COREQ) (30). The COREQ checklist can be viewed in the Supplementary Materials.

## Findings

The participants' characteristics are presented in Table 2. The study sample included four men and 11 women. The education level of the participants ranged from diplomas (*n* = 2) to baccalaureate degrees (*n* = 13). There were six new nurses with <1 year of nursing experience and nine with <2 years. The duration of standardized training in the ICU varied from 1 to 6 months.

**Table 2:** Characteristics of participants in this study (*n* = 15).

**Characteristics**	***N*** **(%) or mean (SD)**
Sex	
Male	4 (26.7)
Female	11 (73.3)
Age	22.13 (0.64)
**Education level**	
Diploma	2 (13.3)
Baccalaureate degree	13 (86.7)
Ethnicity	
Han	14 (93.3)
Tuchia	1 (6.7)
Standardized trained departments	
Internal medicine	3 (20.0)
Emergency department	3 (20.0)
Surgical department	4 (26.7)
Department of obstetrics and gynecology	3 (20.0)
Department of pediatrics	2 (13.3)
Duration of standardized training in the ICU	4.33 (2.44)
Years of experience	
<1	6 (40.0)
<2	9 (60.0)

The findings were identified after analyzing the original data by Colaizzi's methodology. Three themes include: (1) perceived importance; (2) low implementation rate; (3) perceived barriers. The themes are presented in Table 3. The supporting quotations are shown in Table 4. The NGRNs undergoing standardized training in the ICU in China can perceive the importance of EM for MV patients and are actively willing to carry it out. However, they also face many unique challenges and difficulties, resulting in a low implementation rate of clinical EM.

**Table 3:** Main themes and sub-themes categorized from the data.

**Main themes**	**Sub-themes**
Perceived importance	Important and necessary
Low implementation rate	Willing to implement
	Lack of relevant theoretical knowledge and skills
Perceived barriers	Concerns about implementation Risks
	Lack of relevant ICU specialist training
	Overloaded nursing tasks
	Lack of multidisciplinary professional team

**Table 4:** Overview of themes and subthemes and supporting quotations.

**Theme**	**Subthemes**	**Quotations**
Perceived importance	Importance and necessity	“I think it is necessary to do EM for patients. Normal people cannot bear lying in bed all the time, not to mention MV patients. EM can reduce the incidence of complications, such as atelectasis, hypostatic pneumonia, and deep vein thrombosis” (#11). “I remember that my elective college courses included pulmonary rehabilitation for MV patients. EM can reduce the incidence of delirium in ICU patients. I think the sooner EM is implemented, the better the patient outcome will be” (#9). An interviewee also said the muscle strength of patients with mechanical ventilation wound decrease continuously, so it could be necessary to carry out EM, whether passive activities or active activities, the sooner the better (#3).
Low implementation rate	Willing to implement	“I really want to implement EM for patients, but I do not know how to do it, nor dare to do it, let alone do it indiscriminately” (#1). “During the interruption of sedation, I gave the patient a ring rubber band to exercise grip strength” (#2).
	Lack of relevant theoretical knowledge and skills	“When I was assigned to the ICU of Cardiac Surgery, the professional instructor said that EM should be carried out, but did not teach us the detailed methods. We seem to lack professional knowledge in this area” (#12). “Our head nurse is a graduate student. She finds literature on critical care and sends it to us to learn up. We are just trying to do small passive activities for patients” (#6).
Perceived barriers	Concerns about implementation Risks	“Even the MV patients with light sedation have a lot of catheters or monitoring equipment on their bodies. I am afraid of unplanned extubation during EM. The gain is not worth the loss (frowning). EM and nursing safety seem to be contradictory” (#14). “Are there exact entry criteria for early activity? What are the clear contraindications for early activity? What are the contingency plans for accidents during the activity? In fact, our main concern is the safety of patients” (#11). “After the doctor evaluates the patient, the nurse must evaluate the patient again to ensure patient safety. At present, I routinely assess the patient's consciousness, vital signs, and catheters. I am not sure whether the assessment is comprehensive” (#12).
	Lack of relevant ICU specialist training	“The training we have received in EM is too limited. Although we are willing to implement EM, there is no relevant professional technical support. We hope for an increase in relevant post-employment training in the future” (#7). “One of our doctors is very good. As long as he goes to work, he will help all the ICU patients carry out EM. When he does so, I will do it with him. I will be more at ease following him” (#6).
	Overloaded nursing tasks	“I would like to implement EM for patients. But on the one hand, I don't know how to do it, nor dare to do it blindly. On the other hand, I'm really too busy. I have to take care of 3 severe patients alone. In addition to routine nursing work, there are so many documents, nursing assessments, and nursing records. There is no time to implement EM” (#10). “There is no doctor's order to do EM. It is too hasty to complete my own job, and it is too late to implement the early activities” (#13).
	Lack of multidisciplinary professional team	“Sometimes the respiratory therapist will assist us to do EM for the patients, but the number of times is relatively small” (#4). “I think the main obstacle is the lack of EM professionals with leadership. There should be interdisciplinary professional practitioners to coordinate management of EM and realize the sharing of advantageous resources” (#9). “Relying on nurses alone is not enough. Rehabilitation specialists, respiratory therapists, and other interdisciplinary professionals should participate together” (#8). “I think that professionals such as rehabilitation specialists should join the multidisciplinary team for EM, and they can have more professional control in plan formulation, safety assessment and clinical implementation” (#15). “If there is an interdisciplinary team of professionals to participate, I think the EM of mechanically ventilated patients will be better guaranteed in terms of quality and safety” (#5).

*EM, early mobilization; MV, mechanical ventilation*.

### Perceived Importance

With the development of medical technology, the focus of critical care medicine has gradually extended from improving the survival rate of patients to improving prognosis. Early rehabilitation has gradually become a focus in the medical and nursing fields. The NGRNs recognized the importance and necessity of EM for patients with MV.

#### Importance and Necessity

Most interviewees expressed that EM is beneficial for patient recovery. They believed that EM is necessary and should be performed as early as possible. One interviewee stated that EM plays an important role in preventing complications in MV patients. “I think it is necessary to do EM for patients. Normal people cannot bear lying in bed all the time, not to mention MV patients. EM can reduce the incidence of complications, such as atelectasis, hypostatic pneumonia, and deep vein thrombosis” (#11).

Similarly, another interviewee believed that EM is necessary for MV patients and should be done as soon as possible. “I remember that my elective college courses included pulmonary rehabilitation for MV patients. EM can reduce the incidence of delirium in ICU patients. I think the sooner EM is implemented, the better the patient outcome will be” (#9). An interviewee also said the muscle strength of patients with mechanical ventilation wound decrease continuously, so it could be necessary to carry out EM, whether passive activities or active activities, the sooner the better (#3).

### Low Implementation Rate

Although the NGRNs trained in the ICU have a crucial awareness and positive attitude toward EM, new nurses lack clinical experience and expertise pertaining to the ICU and are limited by various obstacles, resulting in inconsistent attitudes and behaviors. The implementation rate of EM in MV patients was relatively low.

#### Willingness to Implement

After recognizing the importance of implementing EM for MV patients, the NGRNs expressed a willingness to implement it in the clinic. “I really want to implement EM for patients, but I do not know how to do it, nor dare to do it, let alone do it indiscriminately” (#1).

Some interviewees reported their experience of implementing EM. However, they focused on helping patients with passive and active activities in bed that hardly involved the patients' standing up or walking out of bed. “During the interruption of sedation, I gave the patient a ring rubber band to exercise grip strength” (#2).

#### Lack of Relevant Theoretical Knowledge and Skills

While the NGRNs expressed a strong desire to implement EM for MV patients, they also mentioned that because of the lack of relevant theoretical knowledge and skills pertaining to EM, they were often in a state of confusion because of not knowing how to implement it in a standardized manner. As a result, the implementation rate of EM in clinical practice is low. “When I was assigned to the ICU of Cardiac Surgery, the professional instructor said that EM should be carried out, but did not teach us the detailed methods. We seem to lack professional knowledge in this area” (#12).

Knowledge acquisition is a prerequisite for nursing practice. Some interviewees can try to carry out EM under training, but there is still a lack of systematic and standardized EM programs. “Our head nurse is a graduate student. She finds literature on critical care and sends it to us to learn up. We are just trying to do small passive activities for patients” (#6).

### Perceived Barriers

Although the NGRNs have recognized the importance and necessity of EM and are willing to implement it in the clinical setting, the clinical implementation rate of EM is low. There may be barriers such as heavy nursing task load, insufficient human resources, and risks that limit the implementation of EM. The NGRNs also expressed a strong desire to strengthen specialty training and the formation of professional multidisciplinary teams for EM.

#### Concerns About Implementation Risks

Most respondents expressed concern about the impact of EM on patient safety. “Even the MV patients with light sedation have a lot of catheters or monitoring equipment on their bodies. I am afraid of unplanned extubation during EM. The gain is not worth the loss (frowning). EM and nursing safety seem to be contradictory” (#14). “Are there exact entry criteria for early activity? What are the clear contraindications for early activity? What are the contingency plans for accidents during the activity? In fact, our main concern is the safety of patients” (#11).

Furthermore, some interviewees said that to ensure safety during EM, it is necessary to master risk assessment methods and take safety risk prevention measures. These pose a greater challenge to NGRNs who are just starting clinical care and lack EM-related knowledge and experience. “After the doctor evaluates the patient, the nurse must evaluate the patient again to ensure patient safety. At present, I routinely assess the patient's consciousness, vital signs, and catheters. I am not sure whether the assessment is comprehensive” (#12).

#### Lack of Relevant ICU Specialist Training

Most interviewees voiced a strong demand for professional training in EM. “The training we have received in EM is too limited. Although we are willing to implement EM, there is no relevant professional technical support. We hope for an increase in relevant post-employment training in the future” (#7). An interviewee also said that they preferred to and could carry out EM under the guidance of experienced medical staff.

Furthermore, an interviewee put forward diversified suggestions for training methods. “During the prevention and control of COVID-19, a lot of our professional knowledge acquisition was carried out online. It would be great if professional knowledge of and training pertaining to EM could also be imparted online. I think, maybe we will do better if the standard process and risk prevention pertaining to EM is more clearly defined through training” (#6).

#### Overloaded Nursing Tasks

The ICU is a highly professional unit and entails a large workload. Moreover, most interviewees stated that heavy nursing task was the main reason for restricting the implementation of EM. “I would like to implement EM for patients. But on the one hand, I don't know how to do it, nor dare to do it blindly. On the other hand, I'm really too busy. I have to take care of 3 severe patients alone. In addition to routine nursing work, there are so many documents, nursing assessments, and nursing records. There is no time to implement EM” (#10). “There is no doctor's order to do EM. It is too hasty to complete my own job, and it is too late to implement the early activities” (#13).

#### Lack of Multidisciplinary Professional Team

Interviewees have expressed their concerns about the lack of professional teams in EM. “Sometimes the respiratory therapist will assist us to do EM for the patients, but the number of times is relatively small” (#4). “I think the main obstacle is the lack of EM professionals with leadership. There should be interdisciplinary professional practitioners to coordinate management of EM and realize the sharing of advantageous resources” (#9).

Many interviewees expressed the need for physicians, rehabilitation therapists, nurses, nutritionists, and other healthscare providers to participate in the practice of EM for MV patients. “Relying on nurses alone is not enough. Rehabilitation specialists, respiratory therapists, and other interdisciplinary professionals should participate together” (#8).

The interviewees fully affirmed the benefits of the multidisciplinary team for the EM. “I think that professionals such as rehabilitation specialists should join the multidisciplinary team for EM, and they can have more professional control in plan formulation, safety assessment and clinical implementation” (#15). “If there is an interdisciplinary team of professionals to participate, I think the EM of mechanically ventilated patients will be better guaranteed in terms of quality and safety” (#5).

## Discussion

This study reveals the knowledge, attitudes, and perceived barriers of Chinese NGRNs undergoing standardized training in the ICU for EM of MV patients. The three themes extracted from the analysis positively supplement post-employment specialist education of NGRNs in the form of recommendations and suggestions. The results of this study show that NGRNs undergoing standardized training in ICUs can recognize the importance and necessity of EM for MV patients. This is consistent with recent research findings (31). With the development of medicine and improvements in technology, the focus of ICU treatment has gradually extended from improving the survival rate to improving the prognosis and quality of life of patients. In this context, EM for MV patients in ICUs is designed to help patients with physical activity as soon as possible in the early stage of mechanical ventilation, to promote patient recovery and prevent complications. Healthcare providers are increasingly concerned about issues regarding the implementation of EM for patients (32).

At the same time, the NGRNs who were part of this study also expressed their willingness to implement EM for MV patients, after acknowledging the importance of EM. This is consistent with the results of Li's study (18). In Wu's research (33), nurses who have been working in the ICU for <5 years had higher attitude scores than nurses in other groups formed by working years. This may be due to the shorter working hours of NGRNs in the ICU. They lack ICU-related specialized knowledge and skills and have less clinical experience. NGRNs are eager to learn, acquire more clinical expertise and improve their professional skills.

However, EM is not carried out as often as necessary. The proportion of patients receiving out of bed activity is about 40.1–60.2%, findings of previous studies show (11, 34). The results of this study show that the NGRNs with fewer working years and little clinical experience lack the knowledge to implement EM for MV patients. This is consistent with the findings of Babazadeh et al. (35). Early rehabilitation is a relatively new viewpoint pertaining to clinical research that has been proposed in recent years. Few studies have been conducted on the early rehabilitation of critically ill patients in China, and the average annual number of articles on the subject published from 2000 to 2018 was only 13 (36). Although evidence-based literature on nursing has been published in recent years (37), owing to the 5-year interval of updating of Chinese textbooks, the teaching of cutting-edge research in the field of critical illness research is insufficient. Every 5 years, China updates the “Outline of the Five-Year Plan and Long-Term Goals for the National Economic and Social Development of the People's Republic of China” (Five-Year Plan). The content of textbooks on critical illness in medical schools is relatively old, and there is a disconnection from clinical needs, which may make NGRNs lack the professional core competence (38).

Safety is a decisive factor for medical personnel to consider whether EM should be implemented. In this study, some NGRNs stated various concerns about issues related to patient safety during the implementation of EM, including unplanned extubation. Moreover, they expressed uncertainty regarding the evaluation of EM. However, a meta-analysis that confirmed the safety of EM showed that the incidence of adverse events was 2.6% (39). Therefore, it is possible that the concerns of NGRNs mainly stem from the lack of knowledge, skills, and experience related to EM.

In this study, NGRNs undergoing standardized training in the ICU also expressed a strong need for ICU-based specialist training related to EM for MV patients. At present, the standardized training of new nurses in the ICU is still mainly focused on basic knowledge and skills, and the development of frontier knowledge of ICU is insufficient. Nursing managers should incorporate EM into standardized training content, disseminate the latest nursing concepts and technologies promptly, and improve the job competencies of NRGNs. In terms of training formats, nursing managers should focus on the learning preferences of the millennial generation of NGRNs, such as the use of the Internet (40). Additionally, experienced tutors of clinical pedagogy can also help new nurses improve the pace of achievement of training goals and clinical nursing capabilities (41).

This study also shows that the burden of ICU-based nursing tasks is also one of the main factors limiting the implementation of EM by NGRNs. This is consistent with the results of previous research (42). Working at ICUs entails concentrated high-precision monitoring and handling of first-aid equipment since different types of critically ill patients are admitted here. These patients have complex and critical illnesses, and their conditions change rapidly. This means that the ICU is a high-tech, labor-intensive, and high-risk medical facility. Therefore, ICU nurses are not only users of advanced equipment, but also the most direct observers of changes in their patients' conditions, as well as the most direct participants in the rescue of critically ill patients. The quality of ICU care is one of the keys to the rescue and survival of ICU patients. This poses a great challenge to NGRNs who are novices in nursing. Moreover, ICU nurses often work night shifts. Their heavy workload, high risk effort, and high-intensity work pressure make them prone to physical and mental fatigue. Additionally, EM has a certain demand for human resources and time, so the implementation of EM in the ICU is subject to certain restrictions. Therefore, hospital managers should rationally allocate ICU nursing human resources, pay attention to the psychological state of ICU nurses, and create a supportive environment to stimulate their work-related enthusiasm and improve and increase the implementation rate of EM (20).

Furthermore, a multi-disciplinary mobile team is also regarded as an effective measure for solving the problem of lower implementation rates of EM due to heavy ICU tasks and a dearth of human resources (43). In this study, the interviewees expressed the urgent need for multidisciplinary EM teams. Multidisciplinary teams (MDT) are strong drivers of clinical implementation and a strong guarantee of continuity of EM. ABCDEF (“A” for Assessment, Prevention, and Manage Pain; “B” for Both Spontaneous Awakening Trials and Spontaneous Breathing Trials; “C” for Choice of Analgesia and Sedation; “D” for Delirium Assess, Prevent, and Manage; “E” for Early Mobility and Exercise; “F” for Family Engagement and Empowerment) is prerequisites for patients to be able to successfully carry out EM, all of which need to be achieved through a multidisciplinary approach (44). In Pan's study (45), physicians, nurses, rehabilitation specialists, nutritionists, and psychotherapists formed a multidisciplinary collaboration team. The results showed that MDT promoted patient recovery and shortened the time taken to perform the out-of-bed activity. Multidisciplinary simulation training for EM should be included in the standardized ICU training of NGRNs. This can help them become familiar with the workflow when working in multidisciplinary teams, clarify the roles and division of labor in each discipline, cultivate the awareness of multidisciplinary cooperation, improve the cooperation ability of NGRNs, and effectively promote the implementation of clinical EM (46).

## Limitations

Although this study provides information on the knowledge, attitudes, and perceived barriers of NGRNs undergoing standardized training in the ICU for EM of MV patients, there are limitations. There could be selection bias as the data were collected only in a third level teaching hospital in Shanghai and lack an overall generalization of findings. Multicentric studies are needed to improve external validity. Also, there is a need to assess the overall knowledge, attitudes, and practice of multidisciplinary healthcare providers.

## Conclusions

This study shows that nurses who are trained in the ICU realize the importance of EM-related knowledge for MV patients. They have good attitudes and positive beliefs about the learning and implementation of EM. However, due to the lack of relevant knowledge and skills, heavier ICU nursing tasks, and other obstacles, the implementation rate of EM needs to be improved. Nursing managers need to strengthen EM training, enrich the training methods, and create a supportive environment in the standardized training of new nurses in the ICU to improve their critical care practice abilities and ensure the quality of critical care.

## Data Availability Statement

The original contributions presented in the study are included in the article/Supplementary Material, further inquiries can be directed to the corresponding authors.

## Ethics Statement

The studies involving human participants were reviewed and approved by the Institutional Review Committee of Shanghai Tenth People's Hospital (No. 2020-KN165-01). The participants provided their written informed consent to participate in this study.

## Author Contributions

JJ and SZ: study design. JJ, SZ, PH, QW, YS, XD, and SY: data collection, data analysis, and manuscript preparation. All authors contributed to the article and approved the submitted version.

## Funding

This work was supported by National Natural Science Foundation of China, 71774117 and Medical Educational Reform Project of Tongji University, 2021YXSZ01. The funding sources provided financial support for the conducts of the research.

## Conflict of Interest

The authors declare that the research was conducted in the absence of any commercial or financial relationships that could be construed as a potential conflict of interest.

## Publisher's Note

All claims expressed in this article are solely those of the authors and do not necessarily represent those of their affiliated organizations, or those of the publisher, the editors and the reviewers. Any product that may be evaluated in this article, or claim that may be made by its manufacturer, is not guaranteed or endorsed by the publisher.
