# Supplementary material for: Knowledge, Attitude, and Perceived Barriers of Newly Graduated Registered Nurses Undergoing Standardized Training in Intensive Care Unit Toward Early Mobilization of Mechanically Ventilated Patients: A Qualitative Study in Shanghai
Source: Front Public Health. 2022 Jan 11;9:802524. doi: 10.3389/fpubh.2021.802524 (PMC8787086; doi:10.3389/fpubh.2021.802524)
Supplement: Supplementary file 1 [file Table_1.docx]

**Table 5**

Consolidated criteria for reporting qualitative studies (COREQ): 32-item checklist

| **No** |  | **Item** | **Guide questions/description** |
| --- | --- | --- | --- |
| **Domain 1: Research team and reflexivity** |  |  |  |
| Personal Characteristics |  |  |  |
| 1. Two researches（first authors） participated, one of them by conducting the face-to-face interviews and the other by recording the participants’ responses, including the non-verbal cues and body language during each interview. |  | Interviewer/facilitator | Which author/s conducted the interview or focus group? |
| 2.3 PhDs in Nursing  3 Masters in Nursing  1BS in Nursing |  | Credentials | What were the researcher's credentials? *E.g. PhD, MD* |
| 3. nurse |  | Occupation | What was their occupation at the time of the study? |
| 4. 6 females & 1 male |  | Gender | Was the researcher male or female? |
| 5.Researchers have all received qualitative research system learning, and all have rich experience in qualitative research. The first author of this article has published ten qualitative research articles. |  | Experience and training | What experience or training did the researcher have? |
| Relationship with participants |  |  |  |
| 6. Researchers build rapport through face-to-face dialogue. |  | Relationship established | Was a relationship established prior to study commencement? |
| 7. reasons for doing the research |  | Participant knowledge of the interviewer | What did the participants know about the researcher? e*.g. personal goals, reasons for doing the research* |
| 8. None |  | Interviewer characteristics | What characteristics were reported about the interviewer/facilitator? e.g. *Bias, assumptions, reasons and interests in the research topic* |
| **Domain 2: study design** |  |  |  |
| Theoretical framework |  |  |  |
| 9. phenomenology |  | Methodological orientation and Theory | What methodological orientation was stated to underpin the study? *e.g. grounded theory, discourse analysis, ethnography, phenomenology, content analysis* |
| Participant selection |  |  |  |
| 10. purposive, convenience |  | Sampling | How were participants selected? *e.g. purposive, convenience, consecutive, snowball* |
| 11. face-to-face |  | Method of approach | How were participants approached? e*.g. face-to-face, telephone, mail, email* |
| 12. 15 |  | Sample size | How many participants were in the study? |
| 13. None |  | Non-participation | How many people refused to participate or dropped out? Reasons? |
| Setting |  |  |  |
| 14. The choice of interview location is based on the preferences and convenience of new nurses, so that the conversation can be confidential. |  | Setting of data collection | Where was the data collected? e*.g. home, clinic, workplace* |
| 15. None |  | Presence of non-participants | Was anyone else present besides the participants and researchers? |
| 16. The characteristics of participants are presented in Table 2. |  | Description of sample | What are the important characteristics of the sample? *e.g. demographic data, date* |
| Data collection |  |  |  |
| 17. The interview questions are based on the theoretical framework of literature and research. Before data collection, a pilot interview with two new nurses was conducted to ensure clarity and identification of any potential problems. The pre-interviews were considered as a test only and not included in analysis. |  | Interview guide | Were questions, prompts, guides provided by the authors? Was it pilot tested? |
| 18. None |  | Repeat interviews | Were repeat interviews carried out? If yes, how many? |
| 19. audio recording |  | Audio/visual recording | Did the research use audio or visual recording to collect the data? |
| 20. √ |  | Field notes | Were field notes made during and/or after the interview or focus group? |
| 21. 58 min (range: 41-70 min) |  | Duration | What was the duration of the interviews or focus group? |
| 22. √ |  | Data saturation | Was data saturation discussed? |
| 23. √ |  | Transcripts returned | Were transcripts returned to participants for comment and/or correction? |
| **Domain 3: analysis and findings**z |  |  |  |
| Data analysis |  |  |  |
| 24. 3 |  | Number of data coders | How many data coders coded the data? |
| 25. √ |  | Description of the coding tree | Did authors provide a description of the coding tree? |
| 26. Themes were derived from the data |  | Derivation of themes | Were themes identified in advance or derived from the data? |
| 27. Colaizzi's seven-step framework was used for data analysis |  | Software | What software, if applicable, was used to manage the data? |
| 28. √ |  | Participant checking | Did participants provide feedback on the findings? |
| Reporting |  |  |  |
| 29. √ |  | Quotations presented | Were participant quotations presented to illustrate the themes / findings? Was each quotation identified? e*.g. participant number* |
| 30. √ |  | Data and findings consistent | Was there consistency between the data presented and the findings? |
| 31. √ |  | Clarity of major themes | Were major themes clearly presented in the findings? |
| 32. √ |  | Clarity of minor themes | Is there a description of diverse cases or discussion of minor themes? |
